# A Novel One-Dimensional Chaotic System for Image Encryption in Network Transmission Through Base64 Encoding

**DOI:** 10.3390/e27050513

**Published:** 2025-05-10

**Authors:** Linqing Huang, Qingye Huang, Han Chen, Shuting Cai, Xiaoming Xiong, Jian Yang

**Affiliations:** 1 School of Advanced Manufacturing, Guangdong University of Technology, Jieyang 522000, China; hlq@gdut.edu.cn; 2 Institute of Collaborative Innovation, University of Macau, Macau, China; 3121009314@mail2.gdut.edu.cn; 3 School of Marine Science and Technology, Shanwei Institute of Technology, Shanwei 516600, China; hanchengdut@gmail.com; 4 School of Integrated Circuits, Guangdong University of Technology, Guangzhou 510006, China; shutingcai@gdut.edu.cn (S.C.); xmxiong@gdut.edu.cn (X.X.); 5 School of Automation, Guangdong University of Technology, Guangzhou 510006, China

**Keywords:** base64 encoding, multiple image encryption, 1D-chaotic system, SHA-256 hash function, plaintext sensitivity, network transmission

## Abstract

Continuous advancements in digital image transmission technology within network environments have heightened the necessity for secure, convenient, and well-suited image encryption systems. Base64 encoding possesses the ability to convert raw data into printable ASCII characters, facilitating excellent stability transmission across various communication protocols. In this paper, base64 encoding is first used in image encryption to pursue high application value in network transmission. First, a novel one-dimensional discrete chaotic system (1D-LSCM) with complex chaotic behavior is introduced and extensively tested. Second, a new multi-image encryption algorithm based on the proposed 1D-LSCM and base64 encoding is presented. Technically, three original grayscale images are constructed as a color image and encoded in base64 characters. To purse high plaintext sensitivity, the original image is input to the SHA-256 hash function and its output is used to influence the generated keystream employed in the permutation and diffusion process. After scramble and diffusion operations, the base64 ciphertext is obtained. Finally, test results derived from comprehensive tests prove that our proposed algorithm has remarkable security and encryption efficiency.

## 1. Introduction

In recent years, continuous advancements in communications technology have led to significant increase in data transmission volumes. Digital images are often used as carriers of information due to their convenience and versatility, making them easy targets for attackers or eavesdroppers. As one of most effective image security techniques, encryption has been widely researched and used to address these vulnerabilities and protect the confidentiality of data.

However, non-chaotic encryption techniques still have disadvantages such as high computational overhead, insufficient key space, and deterministic behavior [1]. Non-chaotic encryption techniques such as AES, DES, and RSA-based approaches face inherent challenges when applied to image data, while block ciphers such as AES (ECB/CBC modes) struggle with redundancy and bulk data in images, leading to inefficiency. Many non-chaotic systems exhibit linearity or periodic behavior, limiting their resistance to brute-force attacks. For instance, 128-bit AES keys may be vulnerable to quantum-assisted attacks, whereas chaotic systems offer exponentially larger key spaces via continuous parameters. Non-chaotic methods lack sensitivity to initial conditions, making them predictable under known/chosen-plaintext attacks. This contrasts sharply with chaotic systems, where microscopic parameter deviations yield entirely divergent ciphertexts.

Image encryption converts a plaintext image into a noise-like image by combining mathematical theories, cryptographical theories, and image processing techniques. The chaotic system features many excellent inherent characteristics, including ergodicity, aperiodicity, and high sensitivity to the initial conditions and control parameters used to generate pseudo-random sequences during encryption [2,3]. For example, Mansouri et al. proposed a novel one-dimensional chaotic map amplifier (1-DCMA) that enhances chaotic behavior, structure and control parameter sensitivity for 1D chaotic maps. This 1-DCMA was then used to generate pseudo-random sequences that could be further processed to disrupt the pixel positions [2]. In [3], the authors employed the random sequence engendered by a chaotic system to dynamically alter and reorganize the original image. Subsequently, synchronous permutation and diffusion procedures were performed on the permuted image to attain the cipher image.

Recently, many chaotic based image encryption algorithms were developed in combination with other techniques to pursue higher security. Compressed sensing techniques involving sparse signal representation, encoding measurements, and reconstruction algorithms have been extensively employed for image encryption, as they can reduce channel occupancy after compression [4,5,6,7,8,9,10,11,12,13,14]. In one case, a new structured measurement matrix was designed to simultaneously compress and encrypt multiple images [4]. Moysis et al. proposed a novel pseudo-random bit generator and an image encryption technique based on shuffling the bit levels [13]. The integration of shuffling and XOR operations produces a ciphertext image that is robust against a variety of attacks. In 2022, Ichraf et al. proposed two new piecewise compound one-dimensional chaotic maps to merge traditional and straightforward one-dimensional chaotic maps [14]. Compared to classical chaotic maps, these 1D maps show better chaotic performances. In [15], the authors employed an encryption algorithm founded upon chaos theory and matrix semi-tensor product theory. Analogous to neural networks, this approach possesses security advanatges for image encryption. Optical information security technology emphasizes the benefits of innate and expeditious parallel processing of large-volume data. This approach differs from the conventional math-centric computer cryptography and information security technology, and is being investigated for image encryption [16,17,18]. In [17], an optical discrete cosine transform-based double random phase encoding (DCT-DRPE) approach was employed for encrypting the initial image, effectuating an enlarged key space and fast encryption. Moreover, image encryption system based on DNA encoding combine the intricacy of biology and algorithms to improve encryption security [19,20,21,22,23]. In [19], the authors proposed a novel image encryption algorithm based on dynamic DNA encoding to enhance plaintext sensitivity and defend against chosen-plaintext attacks. Generation of the keystreams used in the permutation stage was influenced by the statistical properties of the plaintext. To cope with the ever-increasing amount of data transmission, many multi-image encryption (MIE) algorithms have been investigated to exploit the processing power and the parallel architecture of modern computers [24,25,26] as well as to facilitate efficient storage and communication [4,27,28]. The algorithm for parallel image encryption proposed by Song et al. harnesses parallel computing faculties by optimally utilizing extant threads and processor resources to accomplish elevated encryption velocities [24].

Base64 encoding is widely recognized for its ability to convert raw data into printable ASCII characters, facilitating transmission across various communication protocols [29]. Compared with other encoding rules, the transmission of data encoded in base64 provides excellent stability in network environments [30,31]. For example, binary data transmission may introduce the risk of certain binary values being mistaken for control characters, consequently leading to transmission failures. In ASCII encoding, the characters corresponding to ASCII values between 128 and 255 encompass invisible characters, which may produce different decoding results under different encoding rules. On the other hand, base64 encoding offers the ability to convert diverse file formats such as images, audio, and video files into printable texts. This versatility ensures that all computer files can be conveniently encoded as base64 characters, facilitating consistent treatment and transmission of data. In [32], a file encryption system was developed in which the file is first encoded using a base64 algorithm, then encrypted using the well-known advanced encryption standard (AES). In 2021, Selimovic et al. [33] developed a new image steganography algorithm, that included an innovative method for encoding the original image through base64 in order to expand the capacity of steganography information in the image.

In this paper, we seek to overcome several disadvantages of existing one-dimensional chaotic systems, including low complex dynamic behavior, uneven output, and small chaotic range. Inspired by chaotic sine and logistic systems, we construct a new one-dimensional discrete chaotic system called 1D-SLCM. Furthermore, because base64 encoding has characteristics that make it suitable for information transmission, we introduce a multi-image encryption system that uses base64 encoding and our proposed 1D-SLCM system. The overall contributions of the work are summarized as follows:

1. A new discrete chaotic system (1D-SLCM) is developed and its chaotic performance is extensively tested.
2. Base64 encoding is used in image encryption to achieve high application value in network transmission contexts.
3. By employing the proposed 1D-SLCM system and base64 encoding, we construct a multi-image encryption system. A novel plaintext association framework using the SHA-256 hash function is applied to encrypt the original image, helping to enhance plaintext sensitivity. Complete simulations prove the safety and reliability of our system.

The remainder of this paper is organized as follows: Section 2 provides an introduction to base64 encoding and omega networks; Section 3 constructs an XOR mapping for base64 encoding using an N = 64 omega network; Section 4 presents the proposed one-dimensional discrete chaotic system (1D-SLCM); Section 5 details our novel system for encrypting images through base64 encoding; Section 6 provides the simulation and security analysis results of the proposed algorithm; finally, Section 7 summarizes the paper.

## 2. Related Work

### 2.1. Base64 Encoding

Base64 encoding is a secure and reliable encoding process used to convert data from other formats to ASCII format for internet transmission [34]. The base64 encoding table is shown in Table 1. The process of base64 encoding is as follows:

1. Convert the original data into binary form, then organize the data into 6-bit groups.
2. Translate each group into a decimal number ranging from 0 to 63, where each number corresponds to one character in Table 1.
3. In cases where the last group encompasses fewer than six binary numbers, a trailing 0 is added as a suffix to complete the group. Furthermore, if the last group consists of only two or four binary numbers, either one or two “=” symbols are appended to signify padding of the remaining bits with zeros.

The procedure for decoding base64 is the inverse of the encoding procedure.

### 2.2. Omega Network

An omega network is a shuffle-exchange network that uses binary switching for topological connections to achieve arbitrary interconnection between nodes. The composite routing function of an omega network is shown in Equation (1):

(1) $$\begin{array}{c} K_{n}={\left(\sigma_{n}E_{1}\right)}^{n} \end{array}$$

where $E_{1}$ is the binary switch and $\sigma_{n}$ represents the topological connections. It can be observed that $n=log_{2}N$. For each binary switch, we use a bit control signal to judge the state of the switch. When $E_{1}=0$, the binary switch is in the straight state, while when $E_{1}=1$ it is in the exchange state.

**Figure 1:**
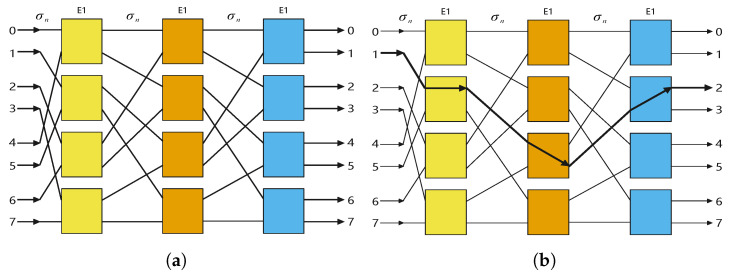
Omega network: (**a**) topology diagram of omega network with N = 8 and (**b**) connection example of omega network with N = 8.

Figure 1 shows the topological diagram of the omega network. Each box in Figure 1 performs switching and has both an exchange state and straight state.

As the connection example of the N = 8 omega network shown in Figure 1b, when the input is set to 1 and the corresponding three control signals $E_{1}$ are configured as 011, the output is 2.

## 3. Mapping Rule for Base64 Encoding

In this section, we use an N = 64 omega network to construct a new mapping rule for base64 encoding. The output port and input ports of the N = 64 omega network each correspond to a base64 character. Each state of the switch unit is controlled by the signal generated by the chaotic system.

## 4. The Proposed New Chaotic System

The sine map and logistic map are both classic one-dimensional chaotic systems. The mathematical expression of the chaotic sine map is provided by Equation (2):

(2) $$\begin{array}{c} x_{n+1}=\frac{1}{4}\gamma sin\left(\pi x_{n}\right). \end{array}$$

When the γ parameter is in the range [3.48,4], the system is in a chaotic state. The logistic map can be expressed as a simple dynamic nonlinear equation:

(3) $$\begin{array}{c} x_{n+1}=\mu x_{n}\left(1-x_{n}\right) \end{array}$$

where $x_{n}\in \left(0,1\right)$ and the μ parameter is in the range from [0,4]. The chaotic logistic system exhibits chaotic behavior when μ is in the range [3.56,4].

Inspired by these two chaotic systems, we propose a new one-dimensional discrete chaotic system called 1D-SLCM that integrates an exponential function $e^{x}$ and cubic nonlinearity term $x^{3}$. The $e^{x}$ term amplifies sensitivity to initial conditions through dynamic phase modulation and range expansion, while the $x^{3}$ term introduces high-order nonlinearity to enrich bifurcation complexity. The proposed 1D-SLCM system is defined by Equation (4):

(4) $$\begin{array}{c} x_{n}=r\times sin\left(\pi e^{x_{n-1}}\left(x_{n-1}^{3}+x_{n-1}+\cos e^{x_{n-1}}+2\right)\right) \end{array}$$

where *r* is a control parameter. To evaluate its performance, we conducted Lyapunov exponent testing, bifurcation analysis, sample entropy analysis, 0–1 testing, and NIST testing.

### 4.1. Lyapunov Exponent Test

**Figure 2:**
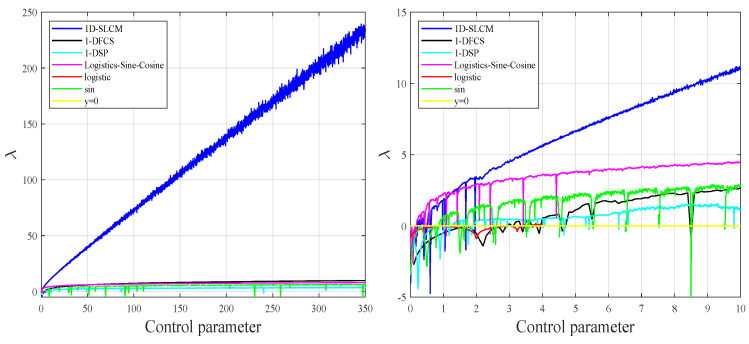
Lyapunov exponent test.

The Lyapunov exponent (LE) reflects the separation rate of adjacent trajectories, serving as an important indicator for judging the chaotic behavior of a system $x_{n+1}=f\left(x_{n}\right)$. The LE is defined by Equation (5):

(5) $$\begin{array}{c} \lambda =\lim_{n\to \infty }\frac{1}{n}\sum_{i=0}^{n-1}\ln\left|f^{\prime }\left(x_{i}\right)\right| \end{array}$$

where *n* is the size of the generated time series $f(x_{n})$. A positive LE score attests to the high sensitivity of initial value, which is an indispensable characteristic in cryptography when pursuing high key sensitivity. Furthermore, a larger LE value indicates a better chaotic property on the part of the corresponding chaotic map. A comparative study of the proposed chaotic map and other chaotic maps, including 1D-SLCM, Logistic–Sine–Cosine [35], 1-DFCS [25], and 1-DSP [36], is presented in Figure 2. As Figure 2 shows, the proposed 1D-SLCM map possesses a LE larger value in a more extensive parameter range than other recently-proposed chaotic systems.

**Figure 3:**
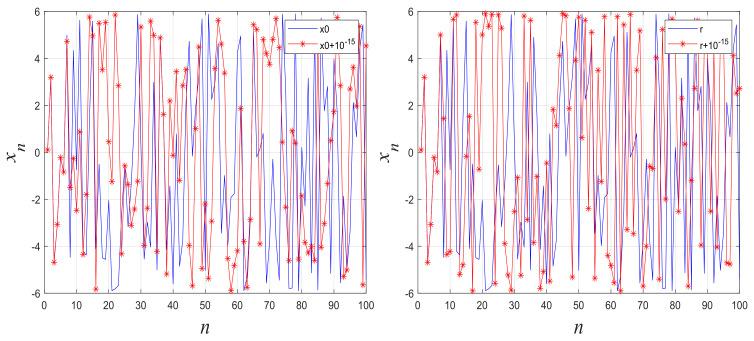
Sensitivity of 1D-SLCM to changes in the initial value and control parameter.

To visually evaluate the initial value and sensitivity of the control parameter in the proposed system, Figure 3 plots output chaotic sequences generated with different initial values or control parameters. The results demonstrate the strong sensitivity of our new chaotic maps to the initial value and control parameter.

### 4.2. Bifurcation Analysis

**Figure 4:**
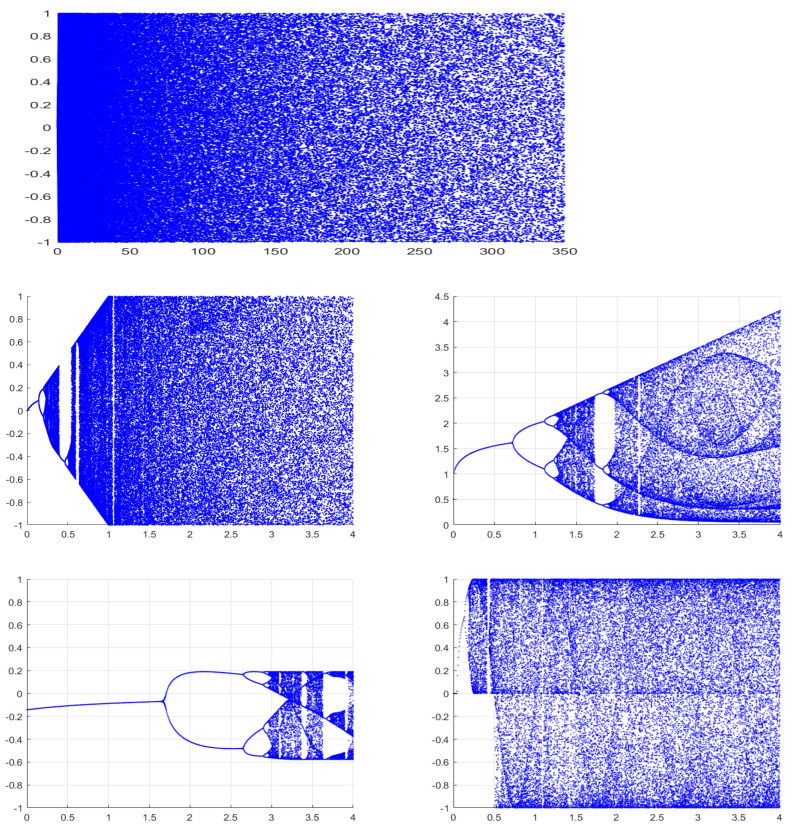
Bifurcation diagram of 1D-SLCM, Logistic–Sine–Cosine, 1-DFCS, and 1-DSP maps.

The long-term behavior of a dynamical system under different control parameter values can be visually plotted by a bifurcation diagram. Bifurcation diagrams of the proposed 1D-SLCM system as well as Logistic–Sine–Cosine, 1-DFCS, and 1-DSP are shown in Figure 4. It can be seen that our new map features a larger chaotic region than the other chaotic systems. In addition, our system avoids the emergence of periodic phases, and its output values possess random distributions.

### 4.3. Sample Entropy

**Figure 5:**
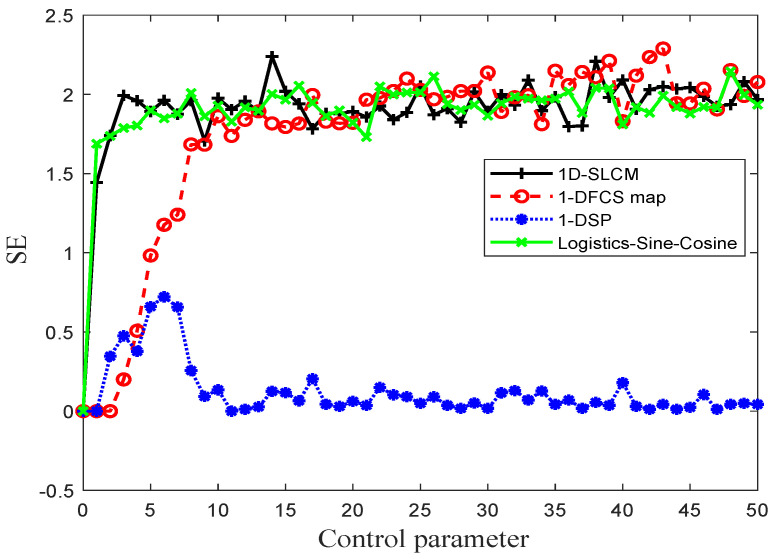
Comparative SE experiment results.

The self-similarity of a time series generated by a dynamical system can be measured using the sample entropy (SE) [37]. Larger SE values indicate lower regularity of the time series and higher complexity of the dynamical system. Here, the template vector $D_{m}\left(i\right)$ is defined in Equation (6), with the size of *m* taken from the time series X(i) to X(i+m−1). The SE of time series *X* is calculated by Equation (7):

(6) $$\begin{array}{c} D_{m}\left(i\right)=\left[X\left(i\right),X\left(i+1\right),\ldots ,X\left(i+m-1\right)\right] \end{array}$$

(7) $$\begin{array}{c} SE\left(m,r,N\right)=-\mathrm{lg}\frac{A}{B} \end{array}$$

where *A* and *B* are the number vectors satisfying $d[X_{m+1}\left(i\right),X_{m+1}\left(j\right)]<r$ and $d[X_{m}\left(i\right),X_{m}\left(j\right)]<r$, respectively, $d[X_{m}\left(i\right),X_{m}\left(j\right)]$ denotes the Chebyshev distance between X(i) and X(i+m−1), and *r* is the given distance. In our experiment, *m* and *r* were chosen as 2 and 0.2×std, respectively. The results of the comparative SE experiment are shown in Figure 5. It can be seen that the time series generated by our proposed 1D-SLCM system have better performance in the SE analysis.

### 4.4. The 0–1 Test

**Figure 6:**
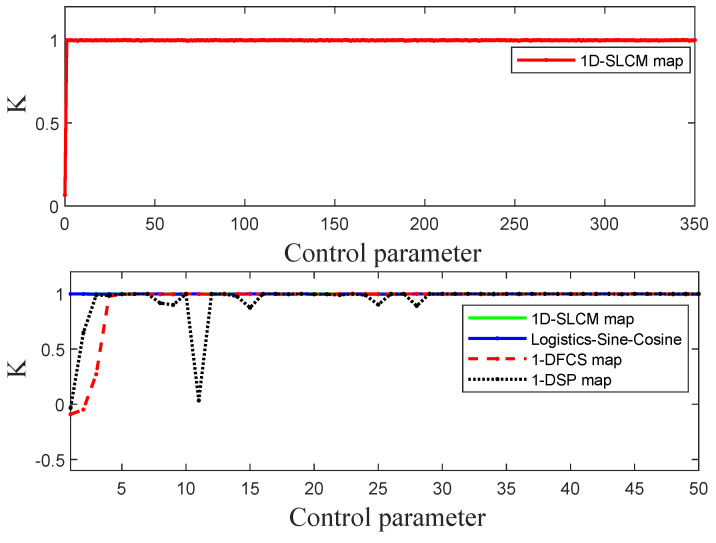
Results of the 0–1 test.

The 0–1 test is employed to identify chaotic phenomena in dynamical systems. The test uses the following equations:

(8) $$\begin{array}{c} K=\frac{\log M(n)}{\log n} \end{array}$$

(9) $$\begin{array}{c} M\left(n\right)=\lim_{N\to \infty }\frac{1}{N}\sum_{i=1}^{n}{\left[p\left(i+n\right)-p\left(i\right)\right]}^{2}+{\left[s\left(i+n\right)-s\left(i\right)\right]}^{2} \end{array}$$

(10) $$\begin{array}{c} p\left(n\right)=\sum_{i=1}^{n}X\left(i\right)\cos ir \end{array}$$

(11) $$\begin{array}{c} s\left(n\right)=\sum_{i=1}^{n}X\left(i\right)\sin ir \end{array}$$

where *X* is a time series with size *N* generated by 1D-SLCM and *r* is a constant that is set as 2 in our experiment. An output result close to 1 means that the dynamic system is in a chaotic state. Figure 6 illustrates the 0–1 test results of four different maps: 1D-SLCM, Logistic–Sine–Cosine, 1-DFCS, and 1-DSP. The K scores for the 1D-SLCM chaotic map are close to 1 when the control parameter r∈(1.4,350), which shows that the proposed 1D-SLCM system is capable of enhancing the dynamic characteristics of traditional one-dimensional chaotic maps.

### 4.5. NIST SP 800-22 Test

To further verify the randomness of the sequence generated by the proposed 1D-LSCM system, we employed the SP 800-22 test developed by the National Institute of Standards and Technology (NIST) as an additional measurement tool. The NIST test suite comprises fifteen subtests. The significance level α was set to the default value of α=0.001. In our test, a total of 125,000 values were generated by the 1D-LSCM system. Each of these values was converted into an integer in (0,255), then into an 8-bit binary number. The transformation operation is shown in Equation (12):

(12) $$\begin{array}{c} X_{i}=x_{i}\times 10^{15}mod\;256 \end{array}$$

where $X_{i}$ and $x_{i}$ are the sequences before and after the transformation, respectively. Therefore, there are 1,000,000 binary numbers in total that form the input of the NIST test. As shown in Table 2, all P-values of the outputs of 1D-LSCM fall within (0.001,1), proving that the sequence iterated by 1D-LSCM possesses the ability to generate sequences with the high randomness required for image encryption.

## 5. The Proposed Encryption Framework

**Figure 7:**
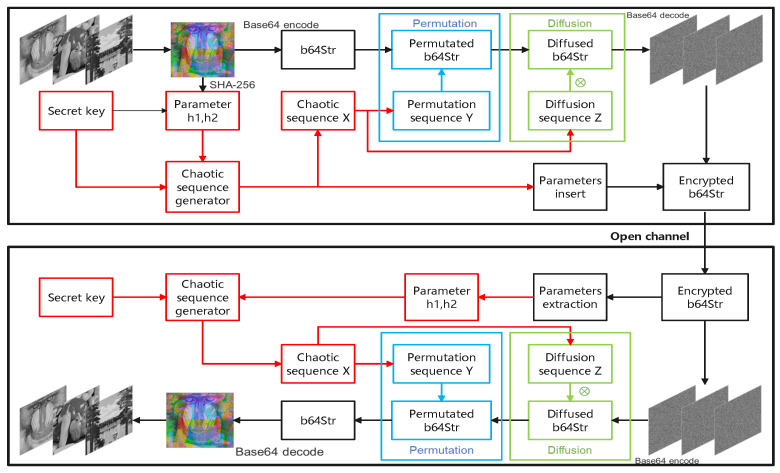
Flow chart of the proposed encryption framework.

This paper proposes a novel image encryption system that leverages the versatility of base64 encoding to convert various forms of files such as text, images, audio, and video into ASCII characters. The flow chart of the proposed encryption framework is shown in Figure 7.

Step 1: Combine three gray images with size M×N into an RGB color image img(M×N×3) where each image corresponds to one channel (red, green or blue).

Step 2: To bolster the sensitivity of the encryption algorithm to plaintext variations, the SHA-256 hash function is employed to derive a 256-bit hash value *h*. We partition *h* into eight distinct groups, as delineated in Equation (13):

(13) $$\begin{array}{c} R_{i}=bin2dec\left(h\left(8\times \left(i-1\right):8\times i\right)\right)\;i=1,2,3\ldots ,8. \end{array}$$

By executing pairwise XOR operations on each group, a pair of parameters $h_{1}$ and $h_{2}$ is produced using Equation (14):

(14) $$\left\{\begin{array}{c} h_{1}=R_{1}⊕R_{2}⊕R_{3}⊕R_{4},\\ h_{2}=R_{5}⊕R_{6}⊕R_{7}⊕R_{8}. \end{array}\right.$$

Step 3: Following Step 1, the RGB color image is subjected to encoding utilizing the base64 technique, yielding a string $b64_{Str}$ with size 4×M×N.

Step 4: In order to expand the key space of the algorithm, eight initial values $x_{i},\;i=1,2,3\ldots ,8$ of the chaotic system are chosen from the key space, then used to iterate the chaotic system $N_{0}+2$ times. The first $N_{0}$ iterations are discarded to obtain eight sequences, as described by Equation (15):

(15) $$\begin{array}{c} x_{i}=\left\{x\left(1\right),\;x\left(2\right),\ldots ,\;x\left(N_{0}+2\right)\right\},\;i=1,2,3,\ldots ,8. \end{array}$$

To improve the key sensitivity of the algorithm, the $N_{0}+2$ element is then modified by Equation (16) before performing subsequent iterations:

(16) $$\left\{\begin{array}{l} x_{i}\left(N_{0}+2\right)=x_{i}\left(N_{0}+2\right)\times \prod_{j=1}^{8}x_{j}\left(N_{0}+2\right)\times h_{1},\;i=1,2,3,4,\\ x_{i}\left(N_{0}+2\right)=x_{i}\left(N_{0}+2\right)\times \prod_{j=1}^{8}x_{j}\left(N_{0}+2\right)\times h_{2},\;i=5,6,7,8. \end{array}\right.$$

Using the eight modified value $x_{i}\left(N_{0}+2\right)\left(i=1,2,3,\ldots ,8\right)$, the chaotic system is continually iterated $\frac{M\times N}{2}$ times to obtain eight sequences, as delineated by Equation (17):

(17) $$\begin{array}{c} X_{i}=\left\{x_{i}\left(N_{0}+3\right),x_{i}\left(N_{0}+4\right),x_{i}\left(N_{0}+5\right),\ldots ,x_{i}\left(N_{0}+\frac{M\times N}{2}\right)\right\},\;i=1,2,3,\ldots ,8. \end{array}$$

Finally, the eight sequences are linked together to obtain the final sequence *X* with size 4×M×N, which is processed using Equation (18) to derive sequence *Y* for permutation and sequence *Z* for diffusion:

(18) $$\left\{\begin{array}{c} X=[X_{1},X_{2},\ldots ,X_{8}],\\ Y=mod(ceil\left(X\times 10^{13}\right)+1,\;4\times M\times N),\\ Z=mod(ceil\left(X\times 10^{13}\right),\;64). \end{array}\right.$$

Step 5: Perform the permutation operation. The base64-encoded string $b64_{Str}$ is scrambled using sequence *Y*, as detailed in Algorithm 1.

Step 6: Perform the diffusion operation. Each character in $b64_{Str}$ is mapped to a new character using *Z* as the control signal, obtaining the encrypted character string $b64_{diffused}$.

Step 7: Because the two parameters $h_{1}$ and $h_{2}$ are needed in the decryption process, $h_{1}$ and $h_{2}$ are both embedded in the ciphertext $b64_{diffused}$ and transmitted to the receiver.

First, $h_{1}$ and $h_{2}$ are each converted into binary sequences, which are integrated to generate a new sequence with a size of 64, denoted as $h_{3}$. Second, two binary numbers $b_{1}$ and $b_{2}$ are generated by $b_{1}=mod\left(x_{1}\left(N_{0}+1\right),1\right),b_{2}=mod\left(x_{1}\left(N_{0}+2\right),1\right)$ and appended to the end of $h_{3}$. Third, the new sequence $h_{3}$ is divided into eleven groups, each of which is converted to a decimal number ranging from 0 to 63. Fourth, each decimal number is mapped to a corresponding base64 character.

Finally, the eleven base64 characters are inserted into $b64_{diffused}$ to obtain the final $b64_{encrypted}$ according to the insertion position $pos_{i}$, which is calculated using Equations (19) and (20):

(19) $$pos=\{x_{2}(N_{0}+1),x_{2}(N_{0}+2),x_{3}(N_{0}+1),x_{3}(N_{0}+2),x_{4}(N_{0}+1),x_{4}(N_{0}+2),\;x_{5}(N_{0}+1),x_{5}(N_{0}+2),x_{6}(N_{0}+1),x_{6}(N_{0}+2),x_{7}\left(N_{0}+1\right)\},$$

(20) $$\begin{array}{c} pos_{i}=mod\left(ceil\left(pos\times 10^{13}\right),n\right),where\;i=1,2,3,\ldots ,11. \end{array}$$

**Algorithm 1** Encryption algorithm**Input:** The base64 encoded string $b64_{Str}$ and two random sequences *Y*, *Z*. **Output:** Encrypted Base64 encoding string $b64_{diffused}$.
1:**for** i=1:4×M×N **do**2:   $temp=b64_{Str}\left(i\right)$3:   $b64_{Str}\left(i\right)=b64_{Str}\left(Y\left(i\right)\right)$4:   $b64_{Str}\left(Y\left(i\right)\right)=temp$5:**end for**6:**for** i=1:4×M×N **do**7:   Perform Base64 encoding diffusion process to obtain $b64_{diffused}$.8:**end for**9:Calculate the sequence $pos_{i}$ with Equation (19) and (20).10:Insert $h_{1}$ and $h_{2}$ to $b64_{diffused}$ and get $b64_{Encrypted}$.


Decryption follows the reverse process to encryption. First, the same random sequence *X* is generated. Second, $h_{1}$ and $h_{2}$ are extracted from the encrypted string $b64_{encrypted}$ to obtain $b64_{diffused}$. Third, sequences *Y* and *Z* are generated by *X* with $h_{1}$ and $h_{2}$ using Equations (15)–(18). Finally, the decryption process is performed as detailed in Algorithm 2.
**Algorithm 2** Decryption algorithm**Input:** The encrypted Base64 encoding string $b64_{diffused}$, sequences *Y*, *Z*.
1:**for** i=1:4×M×N **do**2:   Perform Base64 encoding inverse diffusion process to obtain $b64_{Str}\left(i\right)$.3:**end for**4:**for** i=4×M×N:−1:1 **do**5:   $temp=b64_{Str}\left(i\right)$6:   $b64_{Str}\left(i\right)=b64_{Str}\left(Y\left(i\right)\right)$7:   $b64_{Str}\left(Y\left(i\right)\right)=temp$8:**end for**9:An image img ← Base64 decode $b64_{Str}$.


## 6. Experimental Simulation Results and Security Analysis

**Figure 8:**
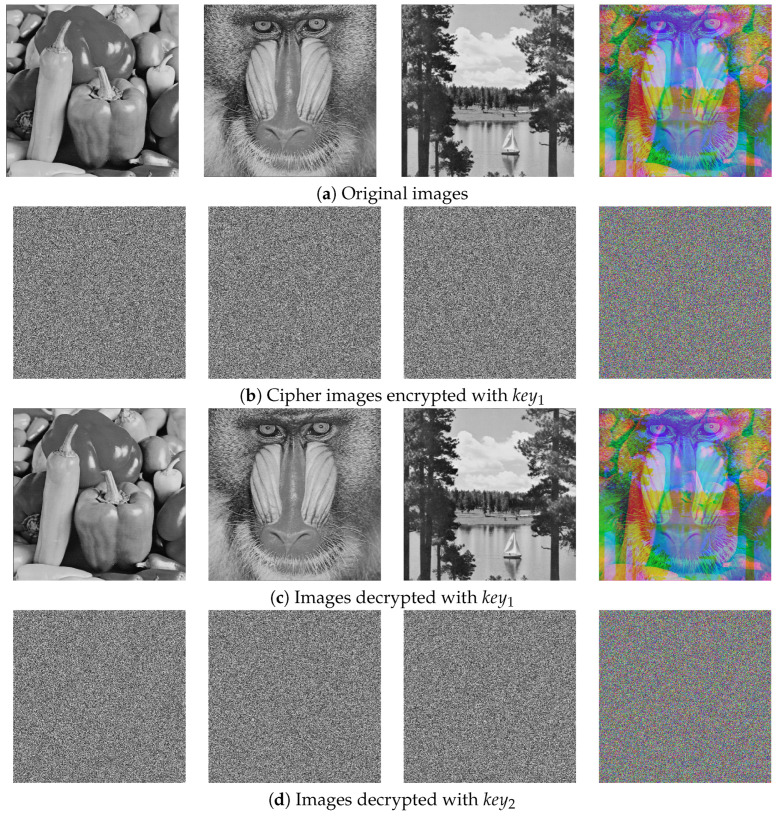
Encrypted and decrypted images.

This section describes various simulations performed with Matlab 2021a software to assess the security and efficiency of the proposed encryption framework. All experiments were conducted on a personal computer with a 2.60 GHz CPU, 16 GB memory, and Windows 11 operating system. It is worth noting that for ease of testing and analysis all encrypted base64 characters were decoded to obtain encrypted images in the spatial domain for further analysis. Figure 8a depicts the three original images and Figure 8b shows the corresponding encrypted images. It can be seen that the encrypted images effectively conceal any information about the original images. Figure 8c,d respectively portray the images when decrypted with the correct key and an incorrect key. It can be seen that the incorrect keys are unable to restore the original images.

### 6.1. Peak Signal-to-Noise Ratio Analysis

The peak signal-to-noise ratio (PSNR) is an objective metric that is commonly used for assessing image quality and dissimilarities between encrypted and original images. When applied to grayscale images, it can be calculated using Equation (21):

(21) $$\left\{\begin{array}{c} PSNR=10\mathrm{lg}(\frac{255\times 255}{MSE})\\ MSE=\frac{\sum_{i=1}^{M}\sum_{j=1}^{N}{\left(P\left(i,j\right)-C\left(i,j\right)\right)}^{2}}{M\times N} \end{array}\right.$$

where *M* and *N* represent the size of the image, while *P* and *C* respectively denote the original and encrypted images. A lower PSNR value signifies a larger difference, indicating a higher level of security achieved by the encryption algorithm. The PSNR values presented in Table 3 indicate that the proposed algorithm yields low PSNR values and better encryption quality.

### 6.2. Plaintext Sensitivity

A reliable encryption algorithm should possess high plaintext sensitivity in order to effectively defend against differential attacks and chosen-plaintext attacks. For this purpose, in our work the original image is input to the SHA-256 hash function and its output is used to influence the generation of the keystream employed in the permutation and diffusion processes. Because the hash algorithm is very sensitive to the input data, the keystreams of two original images that differ by only one pixel are totally different, and the resulting cipher images are entirely distinct. Hence, our algorithm possesses robustness against both differential attacks and chosen-plaintext attacks. To assess the efficacy of encryption in withstanding such attacks, the pixel change rate (NPCR) and unified average changing intensity (UACI) were employed [38]. These metrics are described in Equation (22):

(22) $$\left\{\begin{array}{c} NPCR=\sum_{i=0}^{H}\sum_{j=0}^{W}D(i,j)\times 100\%\\ UACI=\frac{1}{W\times H}\sum_{i=0}^{H}\sum_{j=0}^{W}\frac{\left|c_{1}\left(i,j\right)-c_{2}\left(i,j\right)\right|}{255}\times 100\% \end{array}\right.$$

where $c_{1}$ and $c_{2}$ represent encrypted images derived from two plaintext images that differ by a single pixel and *H* and *W* denote the height and width of the images, respectively. The pixel difference matrix D(i,j) is defined as 0 if $c_{1}\left(i,j\right)=c_{2}\left(i,j\right)$ and 1 if $c_{1}\left(i,j\right)\neq c_{2}\left(i,j\right)$.

We performed experiments using three grayscale images with dimensions of 512×512. The results are presented in Table 4, where it can be seen that the values of NPCR and UACI are both close to their theoretical values.

**Figure 9:**
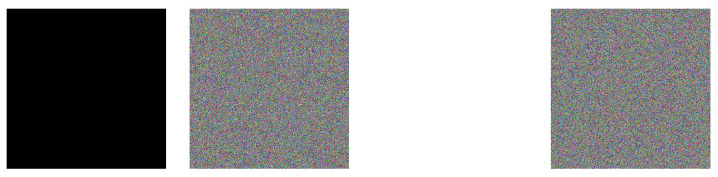
Encryption results for special images.

Pure white and pure black images can typically disable the scrambling operation, and are often used in chosen-plaintext attacks. Here, pure white and pure black images encrypted using our algorithm are presented in Figure 9. Furthermore, the results of NPCR and UACI analysis for the pure white and pure black images are tabulated in Table 5. The above analysis provides compelling evidence of our system’s robustness against differential and chosen-plaintext attacks.

### 6.3. Exhaustive Attack Analysis

#### 6.3.1. Security Key Space

As pointed out in [42], in order to effectively counter brute-force attacks, it is advisable for an encryption algorithm to possess a key space of no less than $2^{100}$. The proposed encryption system encompasses the following set of keys: $key_{xi}\in \left(-5,5\right),i=1,2,3\ldots ,8$ and $N_{0}\in \left(200,2000\right)$ when r=5. We assume that the computer’s accuracy is $10^{15}$ and that the key space is $key_{space}=10^{8}\times {\left(10^{15}\right)}^{8}\times 1800\approx 2^{436}\gg 2^{100}$. This attests to the robustness of our encryption algorithm against brute-force attacks.

#### 6.3.2. Secret Key Sensitivity

**Figure 10:**
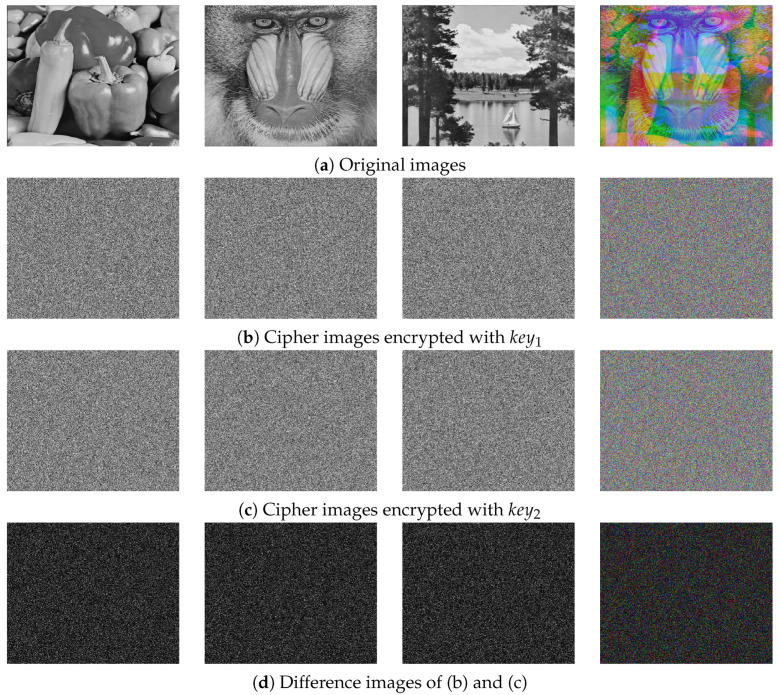
Visual testing of key sensitivity.

In our work, the initial values of 1D-SLCM are used as secret keys. Thus, the keystream experiences significant variations when the image experiences subtle alterations, resulting in significant differences in the cipher image. Figure 10 illustrates encrypted images obtained using two key sets $key_{1}$, $key_{2}$ which have respective differences of $10^{-15}$ in $key_{xi},i=1,2,3\ldots ,8$ and 1 in $N_{0}$. Table 6 presents the corresponding NPCR and UACI test results. It can be seen that even slight changes in the initial value can lead to a completely different encryption result, confirming that the algorithm exhibits a high level of key sensitivity.

### 6.4. Statistical Attack Analysis

#### 6.4.1. Histogram Analysis

**Figure 11:**
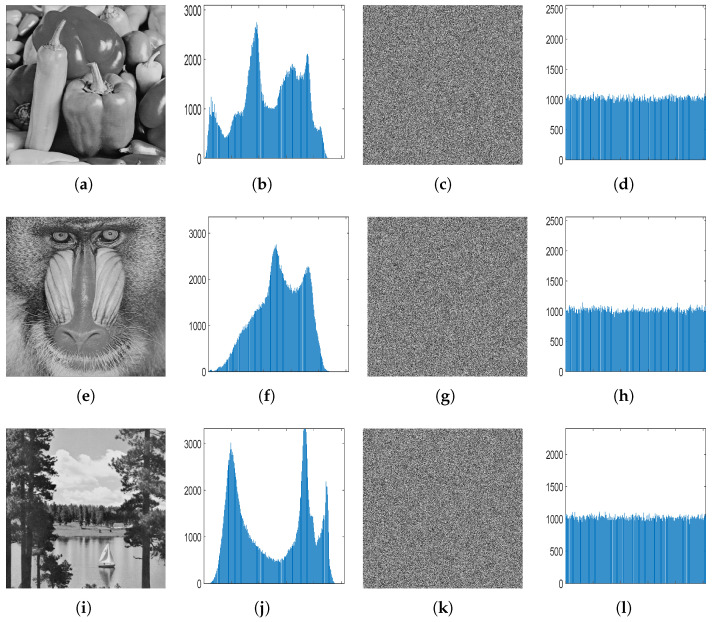
Histogram analysis: (**a**,**e**,**i**) original image, (**b**,**f**,**j**) histograms of the original images, (**c**,**g**,**k**) cipher images, and (**d**,**h**,**l**) histograms of the cipher images.

To enhance resistance against statistical attacks, it is imperative for the histogram of the encryption image to exhibit a flat distribution. By plotting the frequency distribution of different grayscale levels, a flatter histogram is expected to indicate a stronger randomness of pixels in the encrypted image. As shown in Figure 11, the ciphertext image is noise-like, its histogram is evenly distributed, and the frequency of each pixel value is similar, which guarantees the impossibility of an attacker obtaining any useful statistics that could help to infer the original image information.

#### 6.4.2. Correlation Coefficient Test

For every original image, adjacent pixels always exhibit high correlation, which is expected to be destroyed after effective encryption to withstand statistical attacks. The correlation coefficients for a grayscale image are provided by the following formula:

(23) $$r_{xy}=\frac{\mathrm{cov}(x,y)}{\sqrt{D(x)}\times \sqrt{D(y)}}.$$

In Equation (23), $cov\left(x,y\right)=\frac{1}{N}\sum_{i=0}^{N}\left(x_{i}-E\left(x\right)\right)\left(y_{i}-E\left(y\right)\right)$, $D\left(x\right)=\frac{1}{N}\sum_{i=0}^{N}{\left(x_{i}-E\left(x\right)\right)}^{2}$, $E\left(x\right)=\frac{1}{N}\sum_{i=0}^{N}x_{i}$, and x,y represent adjacent pixel values obtained from the four specified directions.

**Figure 12:**
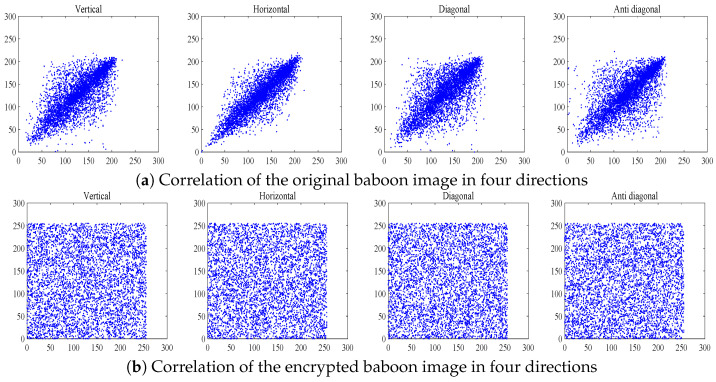
Correlation analysis.

The numerical results of the correlation coefficient are provided in Table 7, showing the good effect of our developed image cryptosystem in eliminating the correlation between adjacent pixels. Furthermore, 5000 pairs of pixels in four directions were selected from both the original and encrypted images. The correlation distribution of these images is plotted in Figure 12. Additionally, a comparison analysis between our scheme and similar algorithms is presented in Table 8. By analyzing Table 8 and Figure 12, it is clear that our encryption algorithm has high effectiveness in eliminating correlation in natural images.

#### 6.4.3. Information Entropy Analysis

The level of information uncertainty was quantified using the information entropy H(m), computed using the formula provided in Equation (24):

(24) $$H\left(m\right)=\sum_{i=0}^{2^{N}-1}p(m_{i})\log\frac{1}{p(m_{i})}$$

where *m* is an information source. For a sufficiently secure image encryption algorithm, the information entropy of a ciphertext image with 256 levels of gray should be close to the theoretical value of 8. Table 9 clearly illustrates that the images encrypted using the proposed technique exhibit entropy values that closely approximate the ideal value.

#### 6.4.4. Cropping and Noise Attacks

**Figure 13:**
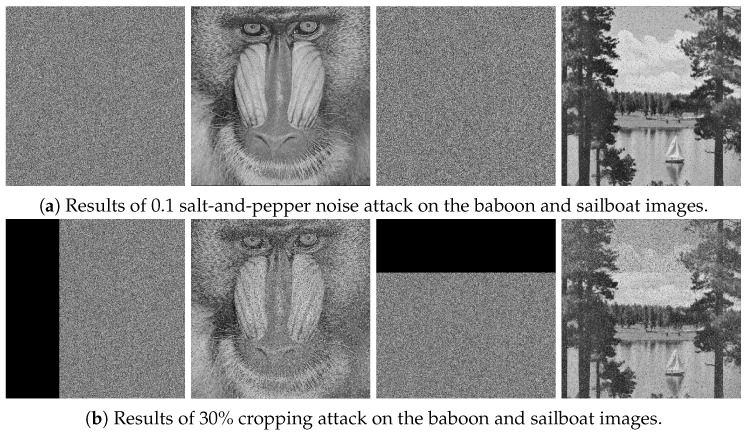
Cropping and noise attack results.

When transmitting encrypted data through public channels, the data may be affected by noise interference or data loss. As can be seen from Figure 13, our algorithm has strong robustness against noise and cropping attacks, meaning that even if the ciphertext image is affected by noise interference or data loss, the main information of the original image can still be restored.

### 6.5. Encryption Time Analysis

Low time complexity is crucial when dealing with resource-constrained platforms or scenarios requiring real-time encryption and/or large-scale data processing. The time complexity of the permutation and diffusion processes is O(MN), meaning that the time complexity of our method is O(MN). We performed 20 runs, with the average values shown in Table 10. Furthermore, Table 10 demonstrates the results of a comparative study of encryption time using the grayscale “Lena” image with different sizes. The proposed algorithm and all tested schemes [39,40,41] used the same platform. The results of this analysis suggest that our encryption algorithm achieves a good balance between security and time complexity, offering a better comprehensive running time performance than other algorithms.

## 7. Conclusions

This paper introduces a new chaotic system called 1D-LSCM along with a novel multi-image encryption algorithm. Simulation results indicate that the proposed algorithm can efficiently encrypt three grayscale images simultaneously. However, due to the characteristics of base64 encoding, this encryption algorithm cannot resist Gaussian attacks, and can only be used for encrypting digital images.

Because all types of computer files, including text, audio, and video, can be converted into base64 encoding, the encryption algorithm proposed in this paper holds significant potential for widespread future adoption in the communications field. The proposed algorithm’s efficiency, safety, and compatibility with various data formats make it a promising solution for secure communication applications. In addiion, its ability to handle different types of data while maintaining high levels of security and efficiency positions it as a reliable choice for ensuring confidentiality in contemporary communication systems.

## Figures and Tables

**Table 1:** Base64 encoding table.

Index	Char	Index	Char	Index	Char	Index	Char
0	A	16	Q	32	g	48	w
1	B	17	R	33	h	49	x
2	C	18	S	34	i	50	y
3	D	19	T	35	j	51	z
4	E	20	U	36	k	52	0
5	F	21	V	37	l	53	1
6	G	22	W	38	m	54	2
7	H	23	X	39	n	55	3
8	I	24	Y	40	o	56	4
9	J	25	Z	41	p	57	5
10	K	26	a	42	q	58	6
11	L	27	b	43	r	59	7
12	M	28	c	44	s	60	8
13	N	29	d	45	t	61	9
14	O	30	e	46	u	62	+
15	P	31	f	47	v	63	/

**Table 2:** NIST test results of 1D-SLCM.

Test Index	*p*-Value	Result
Frequency (Monobit) Test	0.137282	PASS
Frequency Test within a Block	0.090936	PASS
Runs Test	0.657933	PASS
Longest Run of Ones in a Block Test	0.455937	PASS
Binary Matrix Rank Test	0.437274	PASS
Discrete Fourier Transform (Spectral) Test	0.319084	PASS
Non-overlapping Template Matching Test	0.759756	PASS
Overlapping Template Matching Test	0.026948	PASS
Maurers Universal Statistical Test	0.202268	PASS
Linear Complexity Test	0.595549	PASS
Serial Test	0.380485	PASS
Approximate Entropy Test	0.026948	PASS
Cumulative Sums (Cusums) Test	0.739918	PASS
Random Excursions Test	0.468595	PASS
Random Excursions Variant Test	0.437274	PASS

**Table 3:** PSNR values of the three grayscale images.

Image	Pepper	Baboon	Sailboat
PSNR	8.8260	9.5098	8.2186

**Table 4:** Results of the NPCR and UACI analysis.

Image	Proposed		Ref. [39]		Ref. [40]		Ref. [41]	
	NPCR	UACI	NPCR	UACI	NPCR	UACI	NPCR	UACI
Baboon	99.6043	33.4588	99.6032	33.4683	99.6094	33.4625	99.8034	33.4784
Lena	99.6098	33.4606	99.6082	33.4687	99.6098	33.4652	99.7829	33.4873
Sailboat	99.6052	33.4633	99.6099	33.4623	99.6103	33.4621	99.7667	33.4661

**Table 5:** NPCR and UACI analysis for special images.

Image	NPCR	UACI
All-black	99.6082	33.4534
All-white	99.6066	33.4673

**Table 6:** Results of NPCR and UACI analysis of key sensitivity.

		Key1	Key2	Key3	Key4	Key5	Key6	Key7	Key8	$N_{0}$	Average
Baboon	**NPCR**	99.6041	99.6117	99.6093	99.6026	99.6043	99.6133	99.6072	99.6003	99.6047	99.6064
	**UACI**	33.4742	33.4843	33.4741	33.4997	33.4603	33.4744	33.4669	33.4746	33.4656	33.4766
Lena	**NPCR**	99.6094	99.6028	99.6045	99.6021	99.6083	99.6096	99.6127	99.6138	99.6064	99.6084
	**UACI**	33.4761	33.4507	33.4954	33.4608	33.4849	33.4977	33.4608	33.4577	33.4987	33.4734
Sailboat	**NPCR**	99.6131	99.6099	99.6122	99.6072	99.6091	99.6066	99.6069	99.6054	99.6121	99.6092
	**UACI**	33.4771	33.4551	33.4848	33.4938	33.4728	33.4827	33.4542	33.4745	33.4892	33.4835
Fruits	**NPCR**	99.6114	99.6142	99.6109	99.6022	99.6015	99.6135	99.6015	99.6046	99.6117	99.6066
	**UACI**	33.4611	33.4729	33.4541	33.4939	33.4935	33.4711	33.4578	33.4607	33.4960	33.4709
Flower	**NPCR**	99.6121	99.6088	99.6076	99.6029	99.6091	99.6095	99.6129	99.6149	99.6064	99.6093
	**UACI**	33.4836	33.4716	33.4687	33.4965	33.4544	33.4745	33.4876	33.4859	33.4553	33.4738

**Table 7:** Correlation analysis of original images and encrypted images.

Image	Original-Image				Cipher-Image			
	V	H	D	A	V	H	D	A
Pepper	0.9796	0.9787	0.965	0.9733	−0.0094	−0.0222	0.0161	−0.0252
Baboon	0.7454	0.8693	0.7256	0.7206	−0.0066	−0.0168	0.0014	0.0108
Sailboat	0.972	0.9759	0.9548	0.9607	−0.0116	0.0083	−0.0072	0.0225
Mean	-	0.9413	0.8818	0.8849	−0.0092	−0.0102	0.0034	0.0027

**Table 8:** Comparative analysis of correlation coefficients for the encrypted grayscale “Lena” image using various algorithms.

	H	V	D
Proposed	−0.0102	−0.0092	0.0034
Ref. [39]	0.0021	0.0029	0.0023
Ref. [40]	−0.0230	0.0019	−0.0034
Ref. [41]	0.0015	0.0008	−0.0014

**Table 9:** Information entropy of the grayscale images.

Image	Original-Image	Cipher-Image			
		Proposed	Ref. [39]	Ref. [40]	Ref. [41]
Baboon	7.3585	7.9994	7.9994	7.9993	7.9998
Lena	7.4455	7.9991	7.9993	7.9994	7.9998
Sailboat	7.4853	7.9993	7.9992	7.9992	7.9997
Fruits	7.3644	7.9993	7.9995	7.9992	7.9992
Flower	7.4107	7.9992	7.9992	7.9993	7.9993

**Table 10:** Time (in seconds) required for encryption of each grayscale image.

Image Size	Proposed System	Ref. [39]	Ref. [40]	Ref. [41]	Ref. [43]	Ref. [44]
512×512	0.3004	0.3215	0.8562	0.5180	0.0348	0.0934
256×256	0.1229	0.1942	0.2854	0.1439	0.0714	0.1745

## Data Availability

The datasets generated and/or analyzed during the current study are available from the https://www.hlevkin.com/hlevkin/06testimages.htm repository (accessed on 18 April 2025).
